# Adhesive-based protective strategies for enamel cracks during in-office bleaching and their effect on hydrogen peroxide penetration

**DOI:** 10.1007/s00784-026-07200-3

**Published:** 2026-10-03

**Authors:** Letícia Condolo, Laryssa Mylenna Madruga Barbosa, Gabrielle Gomes Centenaro, Taynara S Carneiro, Michael Willian Favoreto, Alessandro D. Loguercio

**Affiliations:** 1 Department of Restorative Dentistry, School of Dentistry, State University of Ponta Grossa, Rua Carlos Cavalcanti, 4748 Bloco M, Sala 64-A, Uvaranas, Ponta Grossa, 84030-900 PR Brazil; 2 Health Sciences Faculty, IDIBO research group, Rey Juan Carlos University, Alcorcón, Madrid, Spain; 3 Department of Restorative Dentistry, School of Dentistry, University of São Paulo, São Paulo, SP Brazil

**Keywords:** Hydrogen peroxide, Tooth fractures, Tooth bleaching, Tooth permeability

## Abstract

**Objectives:**

To evaluate hydrogen peroxide (HP) penetration in teeth with enamel cracks and to investigate if adhesive-based strategies can reduce its permeability without compromising bleaching efficacy (BE).

**Materials and methods:**

Fifty human premolars (*n* = 10 per group) were allocated into five groups: unbleached sound teeth (control), bleached sound teeth, bleached teeth with enamel cracks without adhesive strategy (cracked enamel), and two groups with enamel cracks receiving adhesive protection prior to bleaching (three-step etch-and-rinse adhesive [3-stepER] and universal adhesive used in an etch-and-rinse strategy [UA-ER]). Enamel cracks were detected using transillumination and measured using ImageJ software. In the adhesive groups, phosphoric acid etching was followed by adhesive application before two bleaching sessions. HP penetration into the pulp chamber was quantified using UV–Vis spectrophotometry. Color was assessed at baseline and after 7 and 14 days using digital spectrophotometry. Final bleaching efficacy was calculated from the color change between baseline and day 14 (ΔE_ab,_ ΔE_00_ and ΔWI_D_). Data were analyzed using one-way ANOVA followed by Tukey’s post hoc test (α = 0.05).

**Results:**

HP penetration differed among the bleached groups (*p* < 0.05). The sound group showed the lowest HP levels (0.21 ± 0.06 µg mL⁻¹), whereas the cracked enamel (0.36 ± 0.23 µg mL⁻¹) and 3-stepER (0.32 ± 0.11 µg mL⁻¹) groups showed similar intermediate values. The UA-ER strategy showed the highest HP penetration (1.81 ± 0.34 µg mL⁻¹; *p* < 0.05). No significant differences in BE were observed among the bleached groups.

**Conclusions:**

Neither adhesive-based strategy provided a protective effect against HP diffusion in teeth with enamel cracks. The 3-stepER strategy did not reduce HP penetration compared with unprotected cracked enamel, whereas the UA-ER strategy resulted in significantly greater HP penetration, indicating an adhesive-dependent response.

**Clinical Relevance:**

The use of adhesive systems as a protective strategy during in-office bleaching may not effectively reduce HP diffusion in teeth with enamel cracks. Clinicians should exercise caution when adopting such approaches.

## Introduction

Although several bleaching approaches are available in clinical practice, in-office dental bleaching remains one of the main options due to its controlled application and rapid esthetic outcomes. In this technique, high-concentration hydrogen peroxide (HP) is applied under professional supervision, ensuring greater safety and effectiveness [1, 2]. Once applied to enamel, HP breaks down and releases highly reactive oxygen species that can diffuse through enamel and dentin [3]. These radicals interact with the organic components of the dental substrate, particularly collagen, altering their chemical structure and producing a lighter and more uniform appearance [3, 4].

However, this diffusion capacity is also associated with adverse biological effects. Due to the permeability of the enamel and underlying tooth structure, HP can penetrate the dental tissues and reach the pulp chamber [5], potentially inducing an inflammatory response and leading to bleaching-induced tooth sensitivity (TS) [1, 6–8].

Beyond the intrinsic permeability of dental tissues, structural defects in enamel may further increase tooth permeability by providing additional pathways for the diffusion of bleaching agents [9, 10]. Enamel cracks are linear defects within the enamel structure that may extend along the cervico-incisal or cervico-occlusal directions and may propagate through the tooth [11, 12]. In the literature, superficial cracks confined to enamel are commonly referred to as “craze lines”, whereas the term “enamel microcracks” has also been used to describe microscopic or incomplete fractures of the enamel structure [13, 14]. Their formation has been associated with mechanical and occlusal stresses, trauma, thermal changes, and restorative procedures [14]. In the present study, the term “enamel cracks” was used to describe visible crack lines on the buccal enamel surface identified by transillumination.

Consequently, these defects increase HP permeability and diffusion toward the pulp chamber [9, 10, 15]. Unfortunately, enamel cracks are highly prevalent, with an incidence of up to five cases per 100 individuals per year [13], highlighting the need for effective management strategies for these defects, especially in patients undergoing dental bleaching.

Attempts to limit HP diffusion through these defects using physical protective approaches, such as light-cured barriers, have been unsuccessful, as these materials do not adequately seal enamel cracks or prevent HP permeation [9]. In this clinical context, no ideal sealing material has been identified. Given the lack of effective physical protection, the application of adhesive systems over enamel cracks prior to bleaching may represent a theoretically plausible protective strategy, because of their potential sealing ability.

The rationale for this approach is supported by the well-established ability of adhesive systems to infiltrate enamel microporosities and promote micromechanical interlocking [16]. Accordingly, adhesive resins may penetrate superficial enamel defects, such as enamel cracks, and form a polymer-based sealing layer [17] potentially reducing enamel permeability and limiting HP diffusion toward the pulp chamber. However, given the low molecular weight and high diffusivity of HP [10], whether such an adhesive layer can effectively restrict HP diffusion through enamel cracks remains unclear.

Within this context of biological plausibility but limited experimental validation, evidence regarding the ability of adhesive-based protective strategies to limit HP diffusion remains scarce. Furthermore, it is important to determine whether these strategies influence bleaching efficacy, since reducing HP diffusion may affect esthetic outcomes.

Therefore, the present in vitro study aimed to evaluate HP penetration into the pulp chamber of teeth with enamel cracks and to investigate the effect of two adhesive strategies applied over these defects: three-step etch-and-rinse adhesive (3-stepER) and a universal adhesive used in an etch-and-rinse strategy (UA-ER). Additionally, this study aimed to determine whether the presence of enamel cracks or the application of these adhesive strategies affected bleaching effectiveness. The null hypotheses were that: (1) the application of an adhesive strategy would not affect HP penetration into the pulp chamber; and (2) neither the presence of enamel cracks nor the use of adhesive strategy would affect bleaching efficacy.

## Materials and methods

### Ethical approval 

This *in vitro* study was approved by the Research Ethics Committee of State University of Ponta Grossa (PR/Brazil) under protocol number (8.348.180). A total of 50 human premolars (first and second), extracted for orthodontic or therapeutic reasons, were obtained from the Human Teeth Biobank of the State University of Ponta Grossa (PR/Brazil). The teeth were cleaned and disinfected in a 0.5% chloramine-T solution for 24 h and then stored in artificial saliva at room temperature until use.

### Criteria for teeth selection

Teeth with morphological anomalies detectable by initial visual inspection were excluded. For the control and sound groups, only teeth without enamel cracks or other superficial morphological defects, as assessed under a stereomicroscope at 10× magnification (Lambda LEB-3, ATTO Instruments, Hong Kong, China), were included. For the groups with enamel cracks, only teeth presenting a visible vertical enamel crack on the buccal surface, identified by transillumination, were included. These specimens were subsequently examined under a stereomicroscope at 10× magnification (10× magnification; Lambda LEB-3, ATTO instruments, Hong Kong, China) to identify additional morphological defects, and specimens presenting such defects were excluded.

Additionally, teeth with a Whiteness Index for Dentistry (WID) greater than 25, measured with a digital spectrophotometer (VITA Easyshade Advance 4.0, VITA Zahnfabrik, Bad Säckingen, Germany), were excluded [18]. Teeth were not artificially stained to simulate clinically realistic conditions [19]. Teeth with buccal wall thicknesses < 2.5 mm or > 3.5 mm, assessed by radiographic analysis [20] (Timex 70 C, Gnatus, Ribeirão Preto, SP, Brazil), were also excluded.

### Sample size calculation

The primary objective of this in vitro study was to measure HP concentration into the pulp chamber. Based on a previous study [9], the mean HP concentration into the pulp chamber of teeth with enamel cracks subjected to in-office bleaching was 0.55 ± 0.20 µg mL⁻¹. To detect a 50% difference between groups (either increase or decrease), a two-tailed test was performed with a significance level (α) of 0.05 and a statistical power of 80%.

Based on these parameters, the sample size calculation indicated that nine teeth per group were required. To account for potential specimen loss, one additional tooth was included per group, resulting in a final sample size of 10 teeth per group.

### Detection of enamel cracks

Enamel cracks were identified using transillumination with a VALO curing light (Ultradent, South Jordan, UT, USA) equipped with a Trans Lume lens, positioned perpendicular to the mesial and distal surfaces. Standardized photographic records were obtained using a Canon EOS Rebel Ti3 camera with a 100-mm lens (EF 100 mm f/2.8 L Macro IS USM, Canon, Tokyo, Japan) and a ring flash (Canon MR-14EX, Tokyo, Japan). All photographs were taken under standardized conditions (exposure time: 1/125 s; aperture: f/25; ISO 100; flash in manual mode). The camera-to-subject distance was approximately 15 cm, with a 1:1 magnification ratio, and images were captured in RAW format.

All images were independently evaluated by three calibrated examiners. Both intra- and inter-examiner agreement reached at least 85%, as determined by Kappa statistics. The length of enamel cracks was measured using ImageJ software (National Institutes of Health, Bethesda, MD, USA).

### Randomization and allocation

Teeth presenting enamel cracks were randomly allocated to the experimental groups using block randomization generated in Microsoft Excel. Baseline tooth color was also considered to ensure balanced group distribution. To ensure allocation concealment, the randomization sequence was generated by an independent researcher and implemented using opaque, sealed, sequentially numbered envelopes, which were opened immediately before the experimental procedures. Due to the nature of the interventions, the operator performing the procedures and assessing the outcomes was not blinded to group allocation.

### Experimental groups

Specimens were divided into five experimental groups based on the presence of enamel cracks and the use of an adhesive protective strategy prior to bleaching:

- Control (sound unbleached): Teeth without enamel cracks, with no treatment of the enamel surface.
- Sound (bleached): Teeth without enamel cracks, treated with direct application of a 35% HP bleaching agent (Whiteness HP Maxx, FGM Dental Group, Joinville, SC, Brazil) to the enamel surface.
- Cracked Enamel (CE): Teeth with visible enamel cracks, treated with the same 35% HP bleaching agent (Whiteness HP Maxx, FGM Dental Group, Joinville, SC, Brazil) applied directly to the surface without adhesive protection.
- Cracked Enamel + three-step etch-and-rinse adhesive system (CE+3-stepER): Teeth with enamel cracks were etched with 37.5% phosphoric acid etching (Gel Etchant, Kerr Dental, Orange, CA, USA), followed by application of the three-step etch-and-rinse adhesive system (OptiBond FL, Kerr Dental, Orange, CA, USA) before bleaching with 35% HP (Whiteness HP Maxx, FGM Dental Group, Joinville, SC, Brazil).
- Cracked Enamel + universal adhesive used in an etch-and-rinse strategy (CE+UA-ER): Teeth with enamel cracks were etched with 37.5% phosphoric acid etching (Gel Etchant, Kerr Dental, Orange, CA, USA), followed by application of a universal adhesive system (OptiBond Universal, Kerr Dental, Orange, CA, USA) prior to bleaching with 35% HP (Whiteness HP Maxx, FGM Dental Group, Joinville, SC, Brazil).

### Specimen preparation

The roots were removed approximately 3 mm apical to the cementoenamel junction using a low-speed diamond saw (Isomet 1000, Buehler Ltd, Lake Bluff, IL, USA). After sectioning, the pulp tissue was carefully removed, and the chamber was rinsed with deionized water according to previously established procedures [20]. To improve access to the pulp chamber and ensure sufficient volume for solution collection, a spherical diamond bur was used to enlarge the chamber, creating a cavity capable of holding up to 25 µL of solution, measured with a micropipette.

A radiographic assessment was performed to determine buccal wall thickness. Each specimen was placed in mesial or distal contact with the radiographic film, and images were obtained using an X-ray unit (0.5 s, 70 kVp, 7 mA, and 30-cm focus-to-object distance). The central beam was positioned perpendicular (90°) to the distal crown surface. Digital radiographs were analyzed with New IDA software to precisely measure enamel/dentin thickness [20].

All specimens were stored in artificial saliva (Pharmacy Eficácia, Ponta Grossa, PR, Brazil) composed of carboxymethylcellulose, sodium chloride, potassium chloride, magnesium chloride, dibasic calcium phosphate, glycerin, xylitol, and distilled water [21]. Storage was maintained at 37 °C to simulate intraoral conditions.

### Initial color evaluation

To ensure consistent positioning of the spectrophotometer, individual silicone guides were made for each specimen using a dense violet polyvinyl siloxane silicone (Silic-One, FGM Dental Group, Joinville, SC, Brazil) [22]. A 6-mm circular opening was created at the center of the buccal surface using a punch scalpel to standardize exposure of the middle third of the crown [23].

Color measurements were obtained using a digital spectrophotometer (VITA Easyshade Advance 4.0, VITA Zahnfabrik, Bad Säckingen, Germany). Before each reading, the device was calibrated according to the manufacturer’s instructions and positioned perpendicularly within the standardized window of the guide.

The spectrophotometer provided the L*, a*, and b* coordinates, where L* indicates lightness from 0 (black) to 100 (white), a* represents the green (–) to red (+) axis, and b* represents the blue (–) to yellow (+) axis.

### Obtaining the study calibration curve

All analytical reagents used in this study were of commercial grade and used without further purification. Solutions were prepared with deionized water. To prepare the calibration curve, a 5000 µg mL⁻¹ HP stock solution was obtained by diluting a 35% HP solution (Pharmacy Eficácia, Ponta Grossa, PR, Brazil). This stock was further diluted in acetate buffer (pH 4.0) to generate the required working concentrations, following standard analytical protocols. The effective concentration and quality of the stock solution were verified using a potassium permanganate titration, according to established methods [20].

A series of volumetric dilutions ranging from 0.000 to 1.094 µg mL⁻¹ was prepared. Each solution was placed in a quartz cuvette and analyzed using a Cary 100 UV–Vis spectrophotometer (Varian, Palo Alto, CA, USA) to produce the calibration curve. The absorbance values for the known HP concentrations were used to generate a standard regression line, which served as the reference for interpolating HP concentrations in the specimens. The calibration curve showed a correlation coefficient of *R* = 0.95 (data not shown) [20].

### Treatment protocols and HP penetration into the pulp chamber

In all experimental groups, specimens were removed from artificial saliva storage (Pharmacy Eficácia, Ponta Grossa, PR, Brazil), rinsed with distilled water, and air-dried for 20 s. Each tooth was positioned vertically on a wax base with the occlusal surface facing downward.

Before bleaching, the buccal enamel surface was isolated by a trained operator using a light-cured resin barrier (Top Dam, FGM Dental Group, Joinville, SC, Brazil) to create a standardized 6 × 6 mm window approximately 2 mm thick. Polymerization was performed for 20 s using a light-emitting diode **(**LED) curing unit (Valo Cordless, Ultradent Products, South Jordan, UT, USA) operating in standard mode [24]. All materials were applied according to the manufacturers’ instructions, as described in Table 1.

**Table 1:** Commercial products evaluated in the study, including compositions, and application protocols

Product (Batch number)	Composition	Application (*)
Whiteness HP Maxx 35% [FGM Dental Group, Joinville, SC, Brazil]	35% Hydrogen peroxide, thickener, water, vegetable extracts, sequestrating agents, amide, colorants and glycol.	Mix hydrogen peroxide with the thickening agent in an 18:6 ratio into the mixing container provided with the kit. The gel application period was three times of 15 min, two sessions.
Gel Etchant [Kerr Dental, Orange, CA, USA]	37.5% phosphoric acid in a thixotropic gel formulation with silica fillers and coloring agents.	Apply the etchant for 15 s, rinse thoroughly with water for 15 s, and gently air-dry the surface.
OptiBond FL Primer [Kerr Dental, Orange, CA, USA]	HEMA, GPDM, PAMM, camphorquinone; solvents: ethanol and water.	Apply OptiBond FL Primer to the conditioned surface, actively rubbing for 15 s, and gently air-dry to evaporate the solvent.
OptiBond FL Bond [Kerr Dental, Orange, CA, USA]	Bis-GMA, GDMA, HEMA, TEGDMA, UDMA, camphorquinone; barium glass fillers, ethanol and water.	Apply a thin layer of OptiBond FL Adhesive, gently air-thin, and light-cure for 20 s.
OptiBond Universal [Kerr Dental, Orange, CA, USA]	GPDM, Bis-GMA, HEMA, GDMA, UDMA, TEGDMA, camphorquinone; water-, ethanol-, and acetone-based solvent; silica fillers.	Apply OptiBond Universal to the prepared surface with active brushing for 20 s. Gently air-thin to evaporate the solvent and light-cure for 20 s.

(*) According to the manufacturer

The light guide tip was positioned perpendicular to the surface and maintained in close proximity to the material during photoactivation to ensure adequate energy delivery. The photoactivation time was selected based on previous evidence showing that 20 s provides adequate polymerization of the gingival barrier while maintaining controlled thermal behavior, without excessive temperature increases in the underlying dental tissues [24]. This standardized window ensured uniform exposure to the bleaching agent and reduced variability in HP diffusion. Immediately before the first bleaching session, 25 µL of acetate buffer (pH 4.0) was injected into the pulp chamber to collect all HP diffusing through enamel and dentin during treatment.

For specimens assigned to the 3-stepER strategy (OptiBond FL, Kerr Dental, Orange, CA, USA), the entire exposed 6 × 6 mm enamel window, including the enamel crack, was etched with 37.5% phosphoric acid (Gel Etchant, Kerr Dental, Orange, CA, USA) for 15 s, rinsed for 15 s, and gently air-dried. The primer was applied with a microbrush using active rubbing for 15 s and air-thinned, followed by application of the adhesive over the entire conditioned enamel area. The adhesive was then gently air-thinned and photoactivated for 20 s using the LED curing unit (Valo Cordless, Ultradent Products, South Jordan, UT, USA) operating in standard mode [24].

For specimens treated with the UA-ER strategy (OptiBond Universal, Kerr Dental, Orange, CA, USA) the entire exposed 6 × 6 mm enamel window, including the enamel crack, was etched with 37.5% phosphoric acid (Gel Etchant, Kerr Dental, Orange, CA, USA) for 15 s, rinsed for 15 s, and air-dried. The adhesive was then actively rubbed over the entire conditioned enamel area for 20 s, air-thinned, and photoactivated for 20 s.

For all groups undergoing bleaching, the bleaching gel (Whiteness HP Maxx, FGM Dental Group, Joinville, SC, Brazil) was prepared according to the manufacturer’s instructions and applied to fully cover the standardized buccal area. The bleaching protocol consisted of three consecutive 15-min applications per session, with two sessions at a 7-day interval. HP penetration into the pulp chamber was assessed during the first bleaching session only. Seven days later, after color assessment, the second bleaching session was performed without HP collection. After each session, the gel was removed with gauze, and the surface was rinsed with deionized water.

Immediately after the first bleaching session, the acetate buffer from the pulp chamber was collected with a micropipette and transferred to a glass tube. The pulp chamber was then rinsed four additional times with 25 µL of acetate buffer to ensure maximum HP recovery, and all rinses were pooled.

The resulting 125 µL pooled sample was transferred to a glass tube. Each tube then received 2725 µL of distilled water, 100 µL of 0.5 mg mL⁻¹ leucocrystal violet (Sigma Chemical Co., St. Louis, MO, USA), and 50 µL of 1 mg mL⁻¹ horseradish peroxidase (Peroxidase Type VIA, Sigma Chemical Co.), resulting in a final reaction volume of 3000 µL (3.0 mL). In the presence of HP, this reaction produces a violet chromophore with an absorbance peak at 591 nm, measured using a UV–Vis spectrophotometer (Varian, Palo Alto, CA, USA). Absorbance values were used to calculate HP concentrations (µg mL⁻¹) by interpolation from the calibration curve according to Beer’s Law [20].

### Final color evaluation

Color was assessed at three time points: at baseline, before the first bleaching session; at day 7, seven days after the first bleaching session and immediately before the second bleaching session; and at day 14, seven days after the second bleaching session. After each bleaching session, the bleaching gel was removed, the enamel surface was thoroughly rinsed, and the specimens were stored in artificial saliva (Pharmacy Eficácia, Ponta Grossa, PR, Brazil) for seven days before color assessment. In the adhesive groups, the adhesive layer was not mechanically removed before subsequent color measurements. No surface analysis was performed to determine the integrity or persistence of the adhesive layer after exposure to the bleaching agent and storage in artificial saliva.

Color measurements were performed using a digital spectrophotometer (VITA Easyshade Advance 4.0, VITA Zahnfabrik, Bad Säckingen, Germany). For each measurement, specimens were positioned in their individualized silicone guides to ensure consistent placement of the spectrophotometer tip and measurement reproducibility. Although color was assessed at all three time points, the bleaching efficacy outcomes reported in this study were calculated from the final color change between baseline and day 14.

#### Bleaching efficacy (Color change by ΔE_ab_ and ΔE_00_)

Color change was calculated using two well-established formulas: the CIEL_ab_ color difference (ΔE_ab_) [25]:

$$\:{\varDelta\:E}_{ab}=\:{[{\left({\varDelta\:L}^{*}\right)}^{2}\:+{\left({\varDelta\:a}^{*}\right)}^{2}\:+{\left({\varDelta\:b}^{2}\right)}^{2}\:]}^{1/2}$$

and the CIEDE2000 formula (ΔE_00_) [26]:

$$\:{\varDelta\:E}_{00}=\:{\left[{\left(\frac{\varDelta\:{L}^{{\prime\:}}}{kLSL}\right)}^{2}+\:{\left(\frac{\varDelta\:{C}^{{\prime\:}}}{kCSC}\right)}^{2}+\:{\left(\frac{\varDelta\:{H}^{{\prime\:}}}{kHSH}\right)}^{2}+\:RT\left(\frac{\varDelta\:{C}^{{\prime\:}}\varDelta\:{H}^{{\prime\:}}}{SCSH}\right)\right]}^{1/2}$$

In this formulation, ΔL′, ΔC′, and ΔH′ represent the differences in lightness, chroma, and hue, respectively. The terms SL, SC, and SH correspond to the weighting functions for each component, whereas RT represents a rotation term that accounts for the interaction between chroma and hue differences.

For all analyses, the parametric factors were set to unity (kL = kC = kH = 1), following established recommendations for experimental dental studies [26]. Color changes were considered clinically relevant when exceeding the 50:50% acceptability threshold (2.7 for ΔE_ab_ and 1.2 for ΔE_00_) [27].

#### Whiteness assessment (Whiteness Index for Dentistry)

The Whiteness Index for Dentistry (WI_D_) was calculated to evaluate overall tooth whiteness independently of general color changes [28]:

$$\:{WI}_{D}\:=\:(0.551\:\times\:\:{L}^{*})\:-\:(2.324\:\times\:\:{a}^{*})\:-\:(1.100\:\times\:\:{b}^{*})$$

Changes in WI_D_ (ΔWI_D_) were calculated by subtracting baseline values from post-treatment measurements. A perceptible change was considered clinically relevant when ΔWI_D_ exceeded the 50:50% acceptability threshold (2.6 for ΔWI_D_) [29].

### Statistical analysis

Data analysis began with assessment of normality using the Kolmogorov–Smirnov test and verification of variance homogeneity using the Bartlett test (data not shown). Variables were considered normally distributed and analyzed accordingly.

Baseline variables (buccal thickness, crack length, and WI_D_ values), HP concentration (µg mL⁻¹) in the pulp chamber, and color change parameters (ΔE_ab_, ΔE_00_ and ΔWI_D_) were evaluated using one-way ANOVA, followed by Tukey’s post hoc test for multiple comparisons. Statistical significance was set at α = 0.05. All analyses were performed using SPSS software (version 22.0, IBM Corp., Armonk, NY, USA).

## Results

### Buccal thickness, crack length and baseline WI_D_

No statistically significant differences were observed among the experimental groups for buccal thickness (p = 0.14; Table 2), enamel crack length (p = 0.90; Table 2), or baseline color parameters (p = 0.99; Table 2), as all groups showed comparable values. These findings confirm baseline homogeneity among the groups.

**Table 2:** Means ± standard deviations of buccal thickness, enamel crack length, and baseline WI_D_ for the experimental groups (*)

Groups	Buccal thickness (mm)	Enamel crack length (mm)	Baseline WI_D_
Control	3.1 ± 0.2 A	-	22.5 ± 8.3 ^A^
Sound	3.0 ± 0.3 A	-	21.5 ± 2.3 ^A^
CE	3.2 ± 0.4 A	6.5 ± 4.8 a	21.6 ± 8.2 ^A^
CE + 3-stepER	2.8 ± 0.4 A	6.4 ± 4.5 a	21.7 ± 8.3 ^A^
CE + UA-ER	3.1 ± 0.5 A	6.3 ± 3.7 a	21.8 ± 7.3 ^A^

﻿ Abbreviations: *CE *cracked enamel, *CE+3-stepER *cracked enamel+ three-step etch and rinse adhesive, *CE+UA-ER* cracked enamel + universal adhesive used in an etch-and-rinse strategy

* Different upper and/or lowercase letters superscript or not indicate statistically significant differences among groups within each column (one-way ANOVA; Tukey’s test, p < 0.05)

### HP concentration into the pulp chamber

The HP concentration detected into the pulp chamber differed among the experimental groups (Table 3). No HP was detected in the control group (p < 0.001). Among the bleached groups, the sound enamel group showed the lowest HP concentration (p = 0.01).

The CE group and the CE+3-stepER group showed similar HP concentration (p = 0.07), while the CE+UA-ER group exhibited the highest HP concentration in the pulp chamber (p < 0.001; Table 3).

**Table 3:** Means ± standard deviations of hydrogen peroxide (HP) concentration (µg mL^− 1^) detected into the pulp chamber across experimental groups

Groups	HP concentration (µg mL^− 1^) *
Control	0.00 ± 0.00 A
Sound	0.21 ± 0.06 B
CE	0.36 ± 0.23 C
CE + 3-stepER	0.32 ± 0.11 C
CE + UA-ER	1.81 ± 0.34 D

Abbreviations: *CE *cracked enamel, *CE+3-stepER *cracked enamel+ three-step etch and rinse adhesive, *CE+UA-ER *cracked enamel + universal adhesive used in an etch-and-rinse strategy

*Different uppercase letters indicate statistically significant differences among experimental groups (one-way ANOVA; Tukey’s test, p < 0.05)

### Bleaching efficacy (ΔE_ab_, and ΔE_00_)

Color change parameters after two weeks of bleaching are presented in Table 4. For both ΔE_ab_ and ΔE_00_, all bleached groups showed significantly higher color change than the control group (p < 0.001; Table 4). No significant differences were observed among the bleached groups, regardless of the presence of enamel cracks or the use of adhesive-based protective strategies (p > 0.05). Overall, ΔE_ab_ (p = 0.31; Table 4) and ΔE_00_ (p = 0.16; Table 4) were similar among all bleached groups, indicating comparable bleaching efficacy.

### Whiteness assessment (ΔWI_D_)

For ΔWI_D_, the control group showed lower values than all bleached groups (p < 0.001; Table 4). Among the bleached groups, no significant differences in ΔWI_D_ were observed, regardless of whether a 3-stepER or UA-ER adhesive systems was applied (p = 0.61; Table 4). ΔWI_D_ values were similar across all bleached groups, indicating that adhesive-based protective strategies did not affect bleaching efficacy.

**Table 4:** Means ± standard deviations values of color change parameters after 2 weeks of bleaching for the experimental groups

Groups	ΔE_ab_	ΔE_00_	ΔWI_D_
Control	1.8 ± 1.2 A	1.1 ± 0.7 a	0.0 ± 2.5 ^A^
Sound	7.1 ± 2.4 B	4.4 ± 1.4 b	2.5 ± 6.1 ^B^
CE	4.7 ± 2.8 B	2.7 ± 1.4 b	3.5 ± 5.0 ^B^
CE + 3-stepER	6.8 ± 4.4 B	4.3 ± 2.7 b	4.0 ± 4.5 ^B^
CE + 2- UA-ER	6.9 ± 2.6 B	4.4 ± 1.8 b	1.0 ± 6.2 ^B^

Abbreviations: *CE *cracked enamel, *CE+3-stepER *cracked enamel+ three-step etch and rinse adhesive, *CE+UA-ER *cracked enamel + universal adhesive used in an etch-and-rinse strategy

*Different uppercase letters indicate statistically significant differences among experimental groups (one-way ANOVA; Tukey’s test, p < 0.05)

## Discussion

Enamel cracks are structural discontinuities that increase tooth permeability [30] and facilitate the diffusion of low–molecular weight substances through mineralized tissues, including bleaching agents [9, 10]. In the context of in-office dental bleaching, this increased permeability raises concerns regarding HP diffusion toward the pulp chamber, especially in teeth with structural defects, due to the potential for tooth sensitivity [15, 31]. This concept is supported by the findings of the present study, in which teeth with enamel cracks exhibited higher HP penetration into the pulp chamber compared with sound teeth [9, 10].

Based on the well-established ability of adhesive systems to infiltrate enamel microporosities and promote micromechanical interlocking [3, 32, 33], the application of etch-and-rinse adhesives over enamel cracks has been proposed as a potential sealing strategy to reduce HP diffusion. Acid etching of enamel with phosphoric acid induces a demineralization process that selectively dissolves the mineral phase of the superficial enamel layer and exposes the underlying interprismatic and prismatic structures. As a result, a porous and irregular surface is created, characterized by microporosities that increase surface roughness and surface energy, thereby enhancing micromechanical interlocking with adhesive systems [33, 34].

It is well known that the application of adhesive systems to demineralized enamel provides predictable bond strength and an effective marginal seal in enamel-bonded restorations, mainly due to the formation of tag-like resin extensions within enamel microporosities [32, 34]. Therefore, it was expected that, after adhesive application, the resulting coating would function as a protective barrier against HP diffusion. However, the findings of the present study indicate that HP concentration inside the pulp chamber varied according to the adhesive strategy, with lower HP penetration observed for the 3-stepER than for the UA-ER strategy, supporting the rejection of the first null hypothesis.

A possible explanation for the material-dependent response observed could involve differences in the composition and permeability of the adhesive systems [35]. Simplified adhesives, such as universal adhesive systems, contain predominantly hydrophilic monomers and solvents, which are essential for substrate infiltration but simultaneously increase water sorption and hinder complete polymerization, resulting in a more permeable and less stable polymer network [35, 36].

In contrast, three-step etch-and-rinse adhesives include a separate, more hydrophobic bonding resin layer, typically solvent-free or with fewer hydrophilic components, leading to a higher degree of conversion and reduced permeability [34, 36]. Previous studies have shown that more hydrophilic adhesive interfaces may behave as semi-permeable membranes allowing fluid transudation even after polymerization [34, 36]. Although this mechanism was not directly investigated in the present study, differences in adhesive permeability could potentially influence the diffusion of low-molecular-weight substances such as HP and may partly explain the material-dependent response observed.

Interestingly, when a UA-ER strategy was applied to teeth with enamel cracks, HP levels in the pulp chamber were higher compared with cracked enamel without adhesive application. One factor that could potentially contribute to this finding is the increased enamel permeability induced by phosphoric-acid conditioning [37], which may facilitate HP diffusion through the conditioned enamel. However, phosphoric-acid etching alone cannot explain the greater HP penetration observed with the UA-ER strategy, since the same etching protocol was used with the 3-stepER strategy. Therefore, differences in the composition and application protocols of the adhesive systems, including the separate primer and bonding resin steps of the 3-stepER system, may have contributed to the material-dependent response observed. Because phosphoric-acid conditioning was always followed by adhesive application, the individual contributions of etching and the subsequently applied adhesive layer to HP penetration cannot be distinguished from the present experimental design. Future studies including an acid-etched-only group and adhesive strategies without prior phosphoric-acid conditioning could help clarify the relative contribution of these factors to HP penetration.

Despite the significant differences between adhesive systems, neither strategy provided a protective effect against HP diffusion into the pulp chamber. The 3-stepER strategy resulted in substantially lower HP penetration than the UA-ER strategy; however, HP penetration did not differ significantly from that observed in cracked enamel without adhesive application. Incomplete monomer infiltration could potentially result in deficient hybridization and unfilled interprismatic spaces [38], which might provide diffusion pathways for low–molecular weight substances such as HP. Thus, although not directly investigated, incomplete sealing of the conditioned enamel may be one factor contributing to the inability of the adhesive strategies to restrict HP diffusion, particularly in the presence of enamel cracks [39].

Regarding bleaching efficacy, the second null hypothesis was accepted. Both adhesive-protected groups, regardless of the adhesive strategy employed, showed color changes comparable to those observed in the sound and cracked enamel groups. These findings indicate that neither enamel cracks nor the application of adhesive layers resulted in detectable differences in the bleaching effectiveness outcomes evaluated, suggesting that HP diffusion was sufficient to produce similar whitening outcomes in all bleached groups. This interpretation supported by the HP penetration findings, since neither adhesive strategy reduced peroxide penetration compared with cracked enamel without adhesive application; notably, the UA-ER strategy resulted in even greater HP penetration. Although the adhesive layer was not intentionally removed before color assessment, its integrity and persistence after bleaching and storage in artificial saliva were not evaluated. Therefore, a potential influence of residual adhesive on spectrophotometric measurements cannot be completely excluded. Nevertheless, the absence of significant differences in ΔE_ab_, ΔE_00_, and ΔWI_D_ between adhesive-treated and untreated bleached groups suggests that any potential influence of the adhesive layer did not result in detectable differences in the color-change outcomes evaluated.

The absence of differences in bleaching efficacy among the experimental groups may be explained by the low molecular weight and high diffusibility of HP [3], mainly in teeth with enamel cracks [10]. Even in the presence of an adhesive layer, HP can diffuse through microdefects, areas of incomplete resin infiltration, and interfacial irregularities at the adhesive–enamel interface [3, 32], allowing sufficient penetration to promote effective color change. This is in agreement with the observed HP concentrations in the pulp chamber, reinforcing that the adhesive layer does not effectively limit peroxide diffusion. Consequently, the adhesive layer appears to act mainly as a superficial coating rather than an effective diffusion barrier.

Additionally, a potential contribution of oxidative changes in the adhesive layer induced by HP cannot be excluded. Reactive oxygen species generated during bleaching procedures [3] could potentially alter the polymer network of adhesive systems and thereby affect their permeability to HP. However, this mechanism was not directly evaluated in the present study and should therefore be considered hypothetical. Future studies characterizing the chemical and structural integrity of adhesive layers before and after bleaching could help determine whether bleaching-induced changes contribute to HP diffusion.

Within the scope of the experimental design, some aspects should be considered when interpreting the findings. The adhesive-based protective approach evaluated represents a clinically adopted strategy intended to reduce enamel permeability and minimize potential adverse effects of dental bleaching. The study used a single bleaching gel with an acidic profile, reflecting a commonly used in-office product. Only two commercial adhesive systems were tested, both in the etch-and-rinse strategy. Future studies should evaluate other commercial brands of etch-and-rinse adhesives as alternatives to reduce potential changes in enamel permeability.

Crack characterization was limited to the externally visible surface length, as the methods employed did not allow assessment of crack depth, width, or three-dimensional extension toward dentin. Since these morphological characteristics may influence HP diffusion, future studies using three-dimensional imaging methods, such as X-ray micro-computed tomography, should further investigate their potential effect. Another limitation of the experimental model was the absence of simulated intrapulpal pressure, which may influence HP diffusion under clinical conditions.

Even within these parameters, the results of this study provide relevant insights into the effectiveness of an adhesive-based protective strategy currently used in clinical practice. Although the findings should be interpreted within the experimental conditions and cannot be directly extrapolated to clinical scenarios, they suggest that this approach may not provide the intended protective effect against HP diffusion in enamel with surface cracks. Consequently, this study contributes to a better understanding of whether such strategies effectively balance benefits and potential risks. Future research is needed to explore alternative materials, protocols, and protective approaches that can limit HP diffusion in teeth with enamel cracks without compromising bleaching efficacy, ultimately minimizing potential harm and improving patient comfort.

## Conclusions

The effect of adhesive application on hydrogen peroxide diffusion in teeth with enamel cracks depended on the adhesive strategy used. The three-step etch-and-rinse adhesive did not reduce hydrogen peroxide penetration compared with unprotected cracked enamel, whereas the universal adhesive used in an etch-and-rinse strategy significantly increased hydrogen peroxide penetration. Despite these differences in hydrogen peroxide penetration, bleaching efficacy was not affected.

## Data Availability

No datasets were generated or analysed during the current study.

## References

[1] Loguercio AD, Martins LM, da Silva LM, Favoreto MW, Reis A (2023) In-Office whitening: the latest evidence. tooth whitening: an evidence-based perspective. Springer, pp 159–184

[2] da Silva KL, Favoreto MW, Centenaro GG, Bernardi LG, Borges CPF, Reis A, Loguercio AD (2023) Can all highly concentrated in-office bleaching gels be used as a single-application? Clin Oral Investig 27:3663–3671. 10.1007/s00784-023-04980-w 37017758

[3] Kwon SR, Wertz PW (2015) Review of the Mechanism of Tooth Whitening. J Esthet Restor Dent 27:240–257. 10.1111/jerd.12152 25969131

[4] Eimar H, Siciliano R, Abdallah MN, Nader SA, Amin WM, Martinez PP, Celemin A, Cerruti M, Tamimi F (2012) Hydrogen peroxide whitens teeth by oxidizing the organic structure. J Dent 40(Suppl 2):e25–e33. 10.1016/j.jdent.2012.08.008 22925924

[5] de Oliveira Duque CC, Soares DG, Basso FG, Hebling J, de Souza Costa CA (2017) Influence of enamel/dentin thickness on the toxic and esthetic effects of experimental in-office bleaching protocols. Clin Oral Investig 21:2509–2520. 10.1007/s00784-017-2049-7 28091877

[6] Centenaro GG, Cordeiro DCF, Rodrigues MAM, Lenhani MM, Dias RM, Polo CG, Reis A, Loguercio AD (2025) Efficacy of 35% Self-mixed hydrogen peroxide in in-office bleaching with reduced application time: a single-blind randomized controlled trial. J Dent 106178. 10.1016/j.jdent.2025.106178 41083100

[7] Maran BM, Vochikovski L, de Andrade Hortkoff DR, Stanislawczuk R, Loguercio AD, Reis A (2018) Tooth sensitivity with a desensitizing-containing at-home bleaching gel-a randomized triple-blind clinical trial. J Dent 72:64–70. 10.1016/j.jdent.2018.03.006 29551346

[8] Centenaro GG, Favoreto MW, Carneiro TS, Cordeiro DCF, Loguercio AD (2025) Efficacy and Tooth Sensitivity of Low- Versus High-Concentration Hydrogen Peroxide for In-Office Bleaching: A Randomized Clinical Trial. J Esthet Restor Dent 38:905–914. 10.1111/jerd.70090 PMC13069188 41460823

[9] Favoreto MW, Barbosa LMM, Jiménez-Díez D, Fuentes V, Ceballos L, Reis A, Loguercio AD (2025) Enamel cracks and dental bleaching: does a gingival barrier reduce hydrogen peroxide penetration? J Dent 160:105860. 10.1016/j.jdent.2025.105860 40446901

[10] Briso AL, Lima AP, Goncalves RS, Gallinari MO, dos Santos PH (2014) Transenamel and transdentinal penetration of hydrogen peroxide applied to cracked or microabrasioned enamel. Oper Dent 39:166–173. 10.2341/13-014-L 23802644

[11] Hassan R, Caputo AA, Bunshah RF (1981) Fracture toughness of human enamel. J Dent Res 60:820–827. 10.1177/00220345810600040901 6937518

[12] Abbott P, Leow N (2009) Predictable management of cracked teeth with reversible pulpitis. Aust Dent J 54:306–315. 10.1111/j.1834-7819.2009.01155.x 20415928

[13] Zidane B (2022) Recent Advances in the Diagnosis of Enamel Cracks: A Narrative Review. Diagnostics (Basel) 12:8. 10.3390/diagnostics12082027 PMC9407313 36010379

[14] Dumbryte I, Vailionis A, Skliutas E, Juodkazis S, Malinauskas M (2021) Three-dimensional non-destructive visualization of teeth enamel microcracks using X-ray micro-computed tomography. Sci Rep 11:14810. 10.1038/s41598-021-94303-4 PMC8292334 34285299

[15] Özcan M, Abdin S, Sipahi C (2014) Bleaching induced tooth sensitivity: do the existing enamel craze lines increase sensitivity? A clinical study. Odontology 102:197–202. 10.1007/s10266-013-0104-7 23479310

[16] Buonocore MG (1955) A simple method of increasing the adhesion of acrylic filling materials to enamel surfaces. J Dent Res 34:849–853. 10.1177/00220345550340060801 13271655

[17] Buonocore MG, Matsui A, Gwinnett AJ (1968) Penetration of resin dental materials into enamel surfaces with reference to bonding. Arch Oral Biol 13:61–70. 10.1016/0003-9969(68)90037-x 5237555

[18] Parreiras SO, Favoreto MW, Cruz GP, Gomes A, Borges CPF, Loguercio AD, Reis A (2020) Initial and pulp chamber concentration of hydrogen peroxide using different bleaching products. Braz Dent Sci 23:8. 10.14295/bds.2020.v23i2.1942

[19] Borges AB, Dos Santos KC, da Silva DF, Bottesini VC, Torres C, Mailart MC (2025) Influence of Specimen Pre-staining on in vitro Whitening Efficacy of Hydrogen Peroxide Gels. Oper Dent 50:420–430. 10.2341/24-142-L 40653663

[20] Mena-Serrano AP, Parreiras SO, do Nascimento EM, Borges CP, Berger SB, Loguercio AD, Reis A (2015) Effects of the concentration and composition of in-office bleaching gels on hydrogen peroxide penetration into the pulp chamber. Oper Dent 40:E76–E82. 10.2341/13-352-L 25535786

[21] Cordeiro DCF, Favoreto MW, Centenaro GG, Gumy FN, Loguercio AD, Borges CPF, Reis A (2024) At-home bleaching with carbamide peroxide with concentrations below 10%: bleaching efficacy and permeability in the pulp chamber. Clin Oral Investig 28:224. 10.1007/s00784-024-05604-7 38509406

[22] Santana TR, Silva PFD, Santana MLC, Mattos C, Faria ESAL (2024) Influence of repositioning guides’ color and usage on precision in tooth color measurement with a clinical spectrophotometer. J Appl Oral Sci 32:e20230348. 10.1590/1678-7757-2023-0348 PMC11018295 38537029

[23] Favoreto MW, Madureira MP, Hass V, Maran BM, Parreiras SO, Borges CPF, Reis A, Loguercio AD (2021) A novel carbamide peroxide polymeric nanoparticle bleaching gel: Color change and hydrogen peroxide penetration inside the pulp cavity. J Esthet Restor Dent 33:277–283. 10.1111/jerd.12652 32949453

[24] Favoreto MW, Wendlinger M, Barbosa LMM, Cochinski GD, Oliveira VB, Arrais CAG, Reis A, Loguercio AD (2025) Optimal photoactivation time and thermal behavior of gingival barriers used in in-office dental bleaching. J Dent 160:105888. 10.1016/j.jdent.2025.105888 40516389

[25] De L’Eclairage (1978) CIE Recommendations on uniform color spaces, color-difference equations, psychometric color terms. Color Res Appl 2(1):5–6. 10.1002/j.1520-6378.1977.tb00102.x

[26] Luo MR, Cui G, Rigg BJC (2001) The development of the CIE 2000 colour-difference formula: CIEDE2000. Color Res Appl 26:340–350. 10.1002/col.1049

[27] Paravina RD, Ghinea R, Herrera LJ, Bona AD, Igiel C, Linninger M, Sakai M, Takahashi H, Tashkandi E, Perez Mdel M (2015) Color difference thresholds in dentistry. J Esthet Restor Dent 27(Suppl 1):S1–S9. 10.1111/jerd.12149 25886208

[28] Pérez MM, Ghinea R, Rivas MJ, Yebra A, Ionescu AM, Paravina RD, Herrera LJ (2016) Development of a customized whiteness index for dentistry based on CIELAB color space. Dent Mater 32:461–467. 10.1016/j.dental.2015.12.008 26778404

[29] Pérez MM, Herrera LJ, Carrillo F, Pecho OE, Dudea D, Gasparik C, Ghinea R, Bona AD (2019) Whiteness difference thresholds in dentistry. Dent Mater 35:292–297. 10.1016/j.dental.2018.11.022 30527588

[30] Türp JC, Gobetti JP (1996) The cracked tooth syndrome: an elusive diagnosis. J Am Dent Assoc 127:1502–1507. 10.14219/jada.archive.1996.0060 8908920

[31] Joiner A (2010) Whitening toothpastes: a review of the literature. J Dent 38(Suppl 2):e17–e24. 10.1016/j.jdent.2010.05.017 20562012

[32] Perdigão J (2020) Current perspectives on dental adhesion: (1) Dentin adhesion - not there yet. Jpn Dent Sci Rev 56:190–207. 10.1016/j.jdsr.2020.08.004 PMC8216299 34188727

[33] Breschi L (2025) Buonocore Memorial Lecture 2023: Changing Operative Mindsets with Universal Adhesives and Cements. Oper Dent 50:12–32. 10.2341/24-120-B 39653639

[34] Van Meerbeek B, Yoshihara K, Van Landuyt K, Yoshida Y, Peumans M (2020) From Buonocore’s Pioneering Acid-Etch Technique to Self-Adhering Restoratives. A Status Perspective of Rapidly Advancing Dental Adhesive Technology. J Adhes Dent 22:7–34. 10.3290/j.jad.a43994 32030373

[35] Tay FR, Pashley DH (2003) Have dentin adhesives become too hydrophilic? J Can Dent Assoc 69:726–731 14653938

[36] Cadenaro M, Maravic T, Comba A, Mazzoni A, Fanfoni L, Hilton T, Ferracane J, Breschi L (2019) The role of polymerization in adhesive dentistry. Dent Mater 35:e1–e22. 10.1016/j.dental.2018.11.012 30554830

[37] Kuhar M, Cevc P, Schara M, Funduk N (1997) Enhanced permeability of acid-etched or ground dental enamel. J Prosthet Dent 77:578–582. 10.1016/s0022-3913(97)70098-2 9185049

[38] Knösel M, Bojes M, Jung K, Ziebolz D (2012) Increased susceptibility for white spot lesions by surplus orthodontic etching exceeding bracket base area. Am J Orthod Dentofac Orthop 141:574–582. 10.1016/j.ajodo.2011.11.017 22554751

[39] Ibrahim DFA, Venkiteswaran A, Hasmun NN (2023) The Penetration Depth of Resin Infiltration Into Enamel: A Systematic Review. J Int Soc Prev Community Dent 13:194–207. 10.4103/jispcd.JISPCD_36_23 PMC10411299 37564169

